# Culture Models to Define Key Mediators of Cancer Matrix Remodeling

**DOI:** 10.3389/fonc.2014.00057

**Published:** 2014-03-25

**Authors:** Emily Suzanne Fuller, Viive Maarika Howell

**Affiliations:** ^1^Bill Walsh Translational Cancer Research Laboratory, Kolling Institute of Medical Research, Royal North Shore Hospital, University of Sydney, St. Leonards, NSW, Australia

**Keywords:** high grade serous epithelial ovarian cancer, metastasis, culture models, 3D, synthetic scaffolds

## Abstract

High grade serous epithelial ovarian cancer (HG-SOC) is one of the most devastating gynecological cancers affecting women worldwide, with a poor survival rate despite clinical treatment advances. HG-SOC commonly metastasizes within the peritoneal cavity, primarily to the mesothelial cells of the omentum, which regulate an extracellular matrix rich in collagens type I, III, and IV along with laminin, vitronectin, and fibronectin. Cancer cells depend on their ability to penetrate and invade secondary tissue sites to spread, however a detailed understanding of the molecular mechanisms underlying these processes remain largely unknown. Given the high metastatic potential of HG-SOC and the associated poor clinical outcome, it is extremely important to identify the pathways and the components of which that are responsible for the progression of this disease. *In vitro* methods of recapitulating human disease processes are the critical first step in such investigations. In this context, establishment of an *in vitro* “tumor-like” micro-environment, such as 3D culture, to study early disease and metastasis of human HG-SOC is an important and highly insightful method. In recent years, many such methods have been established to investigate the adhesion and invasion of human ovarian cancer cell lines. The aim of this review is to summarize recent developments in ovarian cancer culture systems and their use to investigate clinically relevant findings concerning the key players in driving human HG-SOC.

High grade serous epithelial ovarian cancer (HG-SOC) is a devastating disease and the most lethal of the gynecological malignancies. Typically treatment consists of surgical debulking, followed by platinum/taxol chemotherapy regimens (1, 2). Treatment fails in up to 70% of patients, and patients with platinum resistant disease have a median survival of 6–12 months (1, 3). Some success has been observed in clinical trials for the palliative management of ascites accumulation using targeted antibody treatment (4), and while this symptom based therapy is clinically important, disease modifying/halting treatments are lacking. Other treatments have shown varied success, including those that target tumor angiogenesis such as bevacizumab alone or in combination with platinum agents and gemcitabine. Many other approaches have been taken including tyrosine kinase inhibitors, angiopoietin inhibitors, histone deacetylase inhibition, and EGF receptor targeting (5). The role of immune cells and interactions with tumor stroma are under intense investigation and may improve the future prospects for immunotherapy based regimes (5). However, response to treatment varies between patients and therefore, the development of personalized care through discovery of predictive molecular or protein markers becomes imperative for effective disease treatment.

Modeling HG-SOC as closely as possible to human disease to facilitate clinically relevant treatment testing is the “holy-grail” in research. A plethora of immortalized ovarian cancer cells and *in vitro* and *in vivo* model systems that utilize these cell lines have been described. Early disease events are arguably the most therapeutically relevant targets of preventative treatments and here, we discuss recently used model systems to identify pathways involved in the development of invasive malignancy.

## Established Epithelial Ovarian Cancer Cell Lines as Model Systems: A Controversial Choice

High grade serous epithelial ovarian cancer has long been thought to arise from the epithelial layer surrounding the ovary (6, 7). However, studies point to a different site of origin, the secretory cells of the fallopian tube fimbria. This highlights the lack of understanding of the histogenesis and molecular signature of this heterogeneous disease (8–14). Anglesio et al. suggested that the biomarker and molecular signatures of ovarian cancer cell lines may be a more accurate and relevant way of grouping “histotypes” over previously determined histological subtypes (15). However, discrepancies between the molecular profile of ovarian cancer cell lines and the tumor types they model have been identified. In fact, these profiles show more similarity between the cell lines themselves, despite differing tissues of origin (8, 16). Further, these reports have raised doubt on the use of a number highly cited ovarian cancer cell lines as models of clinically relevant HG-SOC, in particular A2780 and SKOV3 (8, 15). Cancer cell lines derived from patients who have undergone treatment will represent a population of cells that is intrinsically different from that of the original tumor due to the development of resistance. However, it has been suggested that cell lines derived from untreated tumors are enriched for resistant cells with up-regulation of multi drug resistance associated genes via activation of stress responses during the primary culture process (16).

Immortalized normal ovarian epithelial cells and normal fallopian epithelial cells are increasingly being used to model early stages of cancer development (10, 11, 17–21). While the use of primary cancer cell cultures avoids issues associated with multiple passages (16), this is a labor intensive method, and differences between individual primary cultures leading to lack of reproducibility, may be a significant confounder. Immortalized cell lines offer the advantage of increased stable survival over longer periods in culture and can be manipulated to include many genetic alterations to mimic the disease of interest. Studies using immortalized cells derived from non-transformed normal human fallopian epithelial secretory cells, along with the induction of relevant genetic alterations, have been shown to successfully model human high grade serous cancer biology (10, 11, 19). The use of virally induced immortalization of cells is common; however this may also induce unappreciated effects on tumor development and virally induced tumor initiation is irrelevant to the pathogenesis of ovarian cancer. Non-viral methods using shRNA technology have also successfully targeted relevant genetic factors resulting in transformed cells (11).

Along with the method of cell line derivation, site of origin, and continuous passaging, culture conditions (monolayer, various 3D culture models, organ-like culture models) are also significant effectors of the characteristics of established ovarian cancer cell lines (8, 15, 16, 22). These issues are inherently difficult to address and there is likely no ideal way to completely control for all these changes. To date, particular HG-SOC cell lines have not been reported as being more relevant to 3D culture compared to 2D culture systems. SKOV3 and A2780 are the most commonly cited but may not be the best representations of HG-SOC with their use in 3D likely reflecting their popularity in 2D systems. Therefore at this stage there are no specific criteria for cell line selection for 3D systems and progression from 2D to 3D experiments with the same cell line can be a useful strategy. However, consistent use at a low passage number, of an appropriate cell line to model HG-SOC (via histological and molecular markers) is extremely important.

## *In vitro* Culture Model Systems of HGSEOC

### 2D versus 3D culture methods

Although it is well known that culturing cancer cell lines can drastically alter their genetic characteristics over multiple passages immortalized cancer cell lines remain the gold standard in cancer research and pre-clinical drug testing (22). This is largely because these cell lines display a consistent and relatively homogeneous phenotype over long periods of time, notwithstanding reports of minor side populations with cancer stem-like characteristics in some cell lines (23, 24). Evidence is accumulating that culturing these cells in 3D matrices is far more representative of disease than traditional 2D systems, as they provide structurally similar conditions for cell growth encompassing the ability to manipulate oxygen and growth factor/cytokine gradients as well as the material properties of the matrix (22, 25–30).

**Figure 1:**
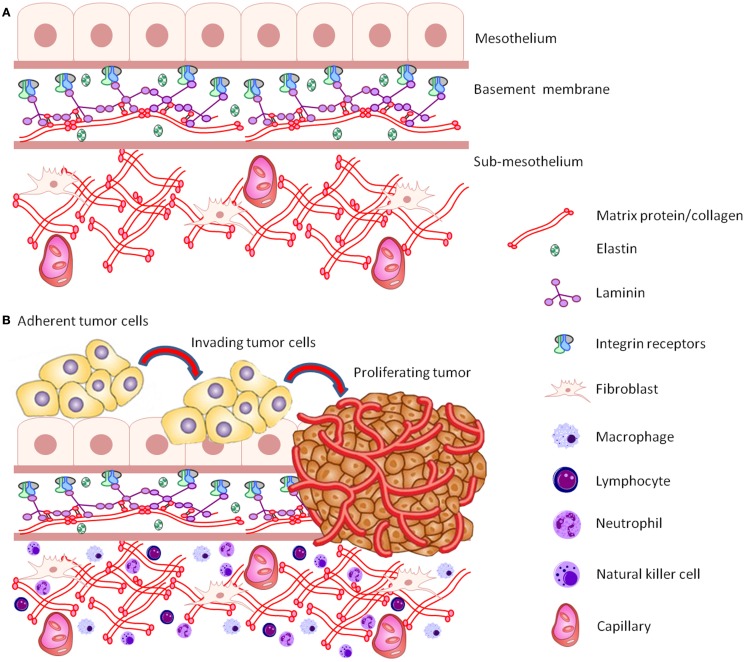
**(A)** Schematic representation of the structure and components of the common peritoneal site of ovarian cancer metastasis. **(B)** Schematic representation of a cluster of adherent ovarian cancer cells invading, proliferating, and destroying basement membrane ECM tissue architecture.

Common methods for assessing ovarian cancer cell proliferation/migration/invasion have included 2D culture growth studies, “scratch” wound healing assays, and penetration through transwell inserts. Scratch wound assays are relatively easy to set up, and very cheap to run and there are now many options for tracking and quantitating cell growth and migration, including the MetaMorph™ and Incucyte™ real-time Imaging systems (31). Migration assays through transwell inserts are more expensive and do not allow for real-time monitoring. Microfluidic assays have the advantage that cells can be grown in controlled chemotactic gradients (31). However, these systems have not to date been utilized widely for ovarian cancer cell culture studies. Cell spreading assays, in which a plastic culture surface is coated with various extracellular matrix (ECM) components (fibronectin or collagen type I) and cells are allowed to spread under serum free conditions for a short period of time, have been used to assess migration of ovarian cancer cells (32). While these methods may provide some useful information regarding the characteristics of certain cancer cell lines and their responses to stimuli (drug treatment, signaling molecules), they lack a 3D micro-environment to accurately mimic pathophysiological conditions. 3D environments containing relevant structural proteins (collagens, laminin, elastin) (Figure 1A), as well as defined tissue organization appropriate to site of tumor growth *in vivo*, are important considerations for recapitulating tumor cell behavior (Figure 1B).

Spread of ovarian cancer cells is complex with cells responding to stimuli from neighboring cells and ECM components and their ability to invade connective tissue is crucial for successful metastasis. In the absence of a requirement for ECM interactions and matrix degradation, 2D systems primarily evaluate the motility of cells, rather than a true invasive barrier removal (29). Care must also be taken when interpreting results based on incomplete 3D representations of a *bona fide* tumor/metastatic site ECM. For example, only a partial understanding of the involvement of proteases/MMPs in the spread and invasion of ovarian cancer cells can be drawn from experiments using matrices that lack structural properties of a relevant ECM. For example, matrigel is substantially less cross-linked and differs in overall composition compared to many tissues (29, 31, 33).

Omental models have been used, in which a primary culture of fibroblasts is grown in 2D with a confluent layer of mesothelial cells grown on top before fluorescently labeled ovarian cancer calls are seeded on a final layer to form a “mock” peritoneal environment. Invasion is typically measured by fluorescent microscopy after the cell layers are cultured in transwell inserts placed over growth promoting media. (26, 34–36). These models provide a more accurate representation of the tissue structure encountered by tumor cells, by supplying a barrier to test “metastatic” invasion of cells in presence of other cells such as fibroblasts that are important to disease processes. However, primary tumor development and the “metastatic cascade” are highly complex processes, and the 2D platforms that are currently used do not typify pathways involved, likely contributing to the unsuccessful translation of findings into *in vivo* systems and eventual failure of many treatments under clinical trial (37).

### Natural versus synthetic 3D platforms

The importance of recapitulating tumor ECM in model systems was highlighted by Infanger and others in their review (25). These authors stated that interactions between tumor cells and their surrounding micro-environment are as pivotal to tumorigenicity as oncogenic mutation (25). Normal homeostatic process and tissue structural properties control the dormancy required after malignant transformation of epithelial cells and when these pathways fail, along with the presence of certain genetic mutations, cells grow uncontrollably and tumors develop (25). Currently, there is a definite lack of studies that evaluate the combined effect of cell–cell, cell–ECM interactions as well as biochemical, biomechanical, and the specific processes that occur during the metastatic processes of ovarian cancer (25, 38).

Hydrogels, such as Matrigel, are commonly used for *in vitro* studies of ovarian cancer cell growth and invasion (29, 32, 39). Other substrates such as collagen gels (40), polyhydroxyethylmethacrylate coated plastics (22), algimatrix, and geltrex are also used to model ECM (16). Natural alternatives include human amniotic membranes (HAM) and chick chorioallantoic membranes (CAM). 3D culture systems incorporating amniotic membranes have been used to assess the spreading and invasive capacities of ovarian cancer cells. These offer the advantage of a physiologically relevant tissue barrier for assessment of cell behavior (41–43). Limitations of these materials are the batch to batch variation, presence of confounding growth factors and other biological components whose effects on culturing experiments are not well known (25, 44). Other non-biological considerations in these model systems, which to date have been largely ignored, are the tissue structural properties as well as gradients of oxygen tension and effects from external physical stimuli (compression, shear stress) (25, 41).

Semi-synthetic matrices such as polyethylene glycol (PEG), hyaluronan, alginate-based, and peptide-based (Puramatrix™) hydrogels are amenable to experimental determination of matrix stiffness and integration of different binding sites and protease cleavage sites (31, 45). Matrix stiffness has been shown to influence endothelial cell behavior independently of matrix molecular composition, highlighting the relevance of matrix material properties in tumor modeling (46). PEG based hydrogels have been used to investigate the role of proteases in the migration of fibroblasts (47) and more recently to investigate cell–ECM interactions and drug resistance of epithelial ovarian cancer cells (48).

Semi-synthetic or synthetic matrices offer the greatest levels of experimental reproducibility due to the control that investigators have in the makeup of the ECM. The study by Loessner et al. is, to date, the most relevant study using a synthetic 3D scaffold to comprehensively investigate ovarian cancer cell growth and response to drugs in an anisotropic biomimetic hydrogel (48). This method enables combination of designed binding sites, protease substrates, other proteins including growth factors and an easily adjustable matrix stiffness. Cells seeded uniformly in the liquid scaffold precursor are exposed to similar levels of biomechanical and biochemical stimuli in all directions (48).

While these models are highly relevant, the addition of other cell types found in the cancer micro-environment (stromal cells, immune cells) would make these models more complete. The immune response has been shown to be clinically relevant in ovarian cancer. Traditionally, immune–cancer cell interactions have been studied in 2D cultures by the addition of immune components or immune stimulatory factors. The establishment of a physiologically relevant tumor micro-environment would enable all cells present (cancer, stromal, immune) to phenotypically resemble those found in disease (49–52). This would create a unique and powerful *in vitro* situation for testing the effects of different immune components and inflammatory responses relevant to disease. For example, TNF-β is known to effect ECM stability, and could therefore influence the capacity of tumor cells to migrate and invade (53). A biologically relevant *in vitro* representation of a tumor is also central for accurately testing drug efficacy, as the interaction of different cell types contributes to the drug response (54). Various 3D models (spheroid cultures, scaffold based 3D cultures, organotypic cultures) would be amenable to the addition of immune factors/cytokines, and although not yet in development, 3D co-culture of many cell types found in ovarian cancer including immune cells should be possible (55, 56).

Heterotypic culture to simulate the micro-environment of ovarian cancer has been shown to be a promising and representative method for investigating stromal–epithelial interactions during disease (57). It has been suggested that modeling ovarian cancer by using 3D cultures of fallopian tube secretory epithelial cells would be more relevant to early stage HG-SOC (58). Combining synthetic matrices, in heterotypic culture with the relevant cells that drive the initiation processes of disease to investigate potential therapeutic targets, would be ideal. A collaborative effort between the NIH, FDA, and the Defense Advanced Research Projects Agency has been instigated to develop and refine methods for functional organ microphysiological systems aimed at drug screening (59). These may also have potential for use in cancer biology. For example, a human liver-like model has been developed to study breast cancer metastases (60). It is possible that such models may, in the future, be adapted to investigate metastases to the liver in ovarian cancer. Table 1 summarizes some of the factors to consider when choosing a method to model cancer cell growth.

**Table 1:** **Summary of factors contributing to the choice of model system for ovarian cancer cell culture**.

	Natural/synthetic	Control of ECM composition	Relevance to *in vivo* tumor	Comments/reference
**COMPONENT/SYSTEM**				
Human amniotic membrane (HAM)	Natural	Low	Medium	Physiologically relevant/provides ECM barrier/batch to batch variation high (42)
Chick chorioallantoic membrane (CAM)	Natural	Low	Medium	Physiologically relevant/provides ECM barrier/batch to batch variation high (43)
Collagen gel (acid extracted type 1 collagen from rat tail)	Synthetic	Medium	Low	Variable ECM stiffness/invasion assessment (binding sites/matrix interaction) (61, 62)
Matrigel (derived from mouse EHS cell secretions; laminin, collagen IV, enactin, various growth factors)	Synthetic	Medium	Low	Widely used (migration and invasion)/batch variation high/irrelevant matrix composition/properties (29, 31, 33)
Alginate/peptide-based (inert polysaccharide, β-d-mannuronic acid, α-l-guluronic acid, calcium ions)	Synthetic	High	Medium	Variable ECM stiffness/defined components/binding sites/matrix interaction (63, 64)
PEG (various cross-linked polyethylene glycol hydrogels) coasted plastics	Synthetic	High	Medium	Variable ECM stiffness/defined components/binding sites/matrix interaction/enzymatically degradable (31, 65)
Heterotypic/organotypic culture	Synthetic	High	High	Relevant micro-environment/cell interaction/combine with synthetic ECM (64, 66)
Spheroid culture	Synthetic	High	Medium	Biologically relevant/cell–cell interactions/combine with synthetic ECM (31, 58, 67)

3D modeling of early stage ovarian cancer, which the aforementioned systems aim to achieve, may be the most relevant for identifying potential targets for disease modifying therapies. The second stage of disease involves the spread of ovarian cancer cells from the primary tumor into the peritoneal space. Experiments to capture the behavior of ovarian cancer cells during metastasis focus on anchorage-independent models of cell migration (68–71). Multicellular aggregate, or spheroid formation is critical for shedding of cancer cells from the primary tumor, and it has recently been shown that the culture of ovarian cancer cells as spheroids in a biomimetic ECM, recapitulates the metastatic niche (72). Further, the biomechanical environment of the peritoneal space plays an important role on cancer cell behavior and spread, and so incorporation of physiological fluid mechanics are appropriate in these systems (41, 69). While the development of oxygen tension gradients limits the size of the multicellular spheroids in culture; it mimics the structure of solid tumors and the potential development of necrotic cores (73, 74). This representation of the physiological micro-environment is relevant and appropriate for the screening of drugs, as penetration into the tumor/spheroid is very different to 2D systems and consequently, the response will also be very different (75). A recent study by Jaeger et al. describes the development of a 3D culture system incorporating an oxygen permeable polymer and micro pillars, to mimic gas delivery via vessels (76). This system offers the potential of larger growth of organotypic models and more realistically represents vascularized tumors *in vivo*.

Tissue chips are a relatively new area of research aimed at incorporating as many components as possible to recapitulate the living tissue and study biological responses to many factors in concert (77, 78). Tissue chips allow the modeling of organ systems in a highly functional and controlled manner. They can incorporate many components relevant to tumor biology such as various 3D matrix components and hydrogels. These systems have the potential as tools for measuring metastatic potential, response to various growth stimulators or inhibitors, immune interactions, and drug responses. However, optimization of parameters such as endpoint data collection is still required in order to use these systems for complex tumor modeling (77, 78).

## Conclusion and Future Perspectives

Many advances have been made in recent years in the development of representative 3D models to mimic ovarian cancer relevant to human HG-SOC. However, these systems are still limited and none to date combine all factors, biomechanical, and biological, to create a complete experimental culture system. This is compounded by recent controversy regarding the molecular characterization of HG-SOC cell lines, with several that are commonly used for research, being shown to be non-representative of this grade of ovarian cancer. It has become clear that when modeling the micro-environment, it is particularly important to create an ECM that closely mimics that relevant to ovarian cancer, and so considerations of the origin of the cell line are important. For example, an ECM relevant to a primary tumor derived cell line may be different from that of a cell line derived from ascites. Likewise, generation of an appropriate ECM for early disease modeling may have different requirements for epithelial cells derived from the fallopian tube to those derived from the ovarian surface. Only through a comprehensive understanding of physiological tumor behavior will it be possible to identify key players in tumor progression, whether these are ECM proteins (MMPs, TIMPs), immune regulators or cytokines or upstream genetic changes in the cancer cells themselves.

While the sophisticated 3D culture models developed in the last few years have circumvented many problems associated with traditional methods, the use of these systems is still in its infancy in part due to the complex nature, cost, and specialized equipment that is often required. Thus these methods are not yet amenable for high-throughput experimentation and pre-clinical testing. However, technological progress in the coming years will hopefully reduce these limitations and see the widespread use of high-throughput screening using 3D culture systems that accurately recapitulate the tumor micro-environment.

## Conflict of Interest Statement

The authors declare that the research was conducted in the absence of any commercial or financial relationships that could be construed as a potential conflict of interest.
